# Caprylic acid suppresses inflammation via TLR4/NF-κB signaling and improves atherosclerosis in ApoE-deficient mice

**DOI:** 10.1186/s12986-019-0359-2

**Published:** 2019-06-06

**Authors:** Xinsheng Zhang, Changyong Xue, Qing Xu, Yong Zhang, Huizi Li, Feng Li, Yinghua Liu, Changjiang Guo

**Affiliations:** 1 Department of Nutrition, Tianjin Institute of Environmental & Operational Medicine, Tianjin, 300050 China; 2 Department of Nutrition, Chinese PLA General Hospital, 28 Fuxing Road, Beijing, 100853 China; 3 Department of Nutrition, PLA Rocket Force Characteristic Medical Center, Beijing, 100088 China; 4 Department of Nutrition, Air Force Medical Center, PLA, Beijing, 100142 China

**Keywords:** Caprylic acid, Inflammation, TLR4, Atherosclerosis, apoE-deficient mice

## Abstract

**Background:**

As reported previously by our group, medium-chain triglycerides can ameliorate atherosclerosis. Given that TLR4 is closely related to atherosclerosis, we hypothesized herein that caprylic acid (C8:0) would suppress inflammation via TLR4/NF-κB signaling and further promote the amelioration of atherosclerosis in apoE- deficient (apoE^−/−^) mice.

**Methods:**

Fifty 6-week male apoE^−/−^ mice were randomly allocated into five diet groups: a high-fat diet (HFD) without or with 2% caprylic acid (C8:0), capric acid (C10:0), stearic acid (C18:0), or linolenic acid (C18:3). RAW246.7 cells were treated with caprylic acid (C8:0), docosahexenoic acid (DHA), palmitic acid (C16:0), and lipopolysaccharide (LPS) with or without TLR4 knock-down (TLR4-KD). The serum lipid profiles, inflammatory biomolecules, and mRNA and protein expression levels were measured. Atherosclerotic lesions that occurred in the aorta and aortic sinuses were evaluated and quantified.

**Results:**

Our results indicated that C8:0 reduced body fat, improved the lipid profiles, suppressed inflammatory cytokine production, downregulated aortic TLR4, MyD88, NF-κB, TNF-α, IKKα, and IKKβ mRNA expression, and alleviated atherosclerosis in the apoE^−/−^ mice (*P* < 0.05). In RAW 264.7 cells, C8:0 diminished the inflammatory response and both mRNA and protein expression of TLR4, MyD88, NF-κB, and TNF-α compared to those in the LPS and C16:0 groups (*P* < 0.05). However, in the TLR4-KD RAW 264.7 cells, C8:0 significantly upregulated NF-κB mRNA and protein expression compared to those in the C16:0 and DHA groups.

**Conclusions:**

These results suggest that C8:0 functions via TLR4/NF-κB signaling to improve the outcomes of apoE^−/−^ mice through suppressing inflammation and ameliorating atherosclerosis. Thus, C8:0 may represent as a promising nutrient against chronic inflammatory diseases.

**Electronic supplementary material:**

The online version of this article (10.1186/s12986-019-0359-2) contains supplementary material, which is available to authorized users.

## Introduction

Atherosclerosis is a chronic inflammatory disease that is characterized by lipid accumulation, smooth muscle cell proliferation, cell apoptosis, necrosis, fibrosis, and local inflammation [1, 2]. Fatty acids have been deemed important dietary factors that affect the occurrence and development of this condition, and different aliphatic acids can contribute to inflammation and atherosclerosis via diverse pathways. Pro-inflammatory genres, such as saturated fatty acids (SFAs) [3, 4] and n-6 polyunsaturated fatty acids (PUFAs), can induce atherosclerosis, whereas n-3 fatty acids that are rich in fish oil, such as docosahexaenoic acid (DHA) and eicosapentaenoic acid (EPA), can inhibit many aspects of inflammation, including leucocyte chemotaxis, adhesion molecule expression and leucocyte-endothelial adhesive interactions [5, 6]. These effects of n-3 fatty acids can lower the morbidity of atherosclerosis and contribute to the prevention of cardiovascular disease (CVD) -related complications [7].

Medium-chain fatty acids (MCFAs), such as caprylic acid (C8:0), capric acid (C10:0), and lauric acid (C12:0), carry a backbone chain with 8 to 12 carbon atoms. MCFAs, in the form of medium-chain triglycerides (MCTs) that are present in milk fat, palm oils, coconuts, and cuphea seed oils [8, 9]. MCFAs have been increasingly noted to be quite different from long-chain fatty acids (LCFAs) both physically and metabolically, although they both belong to the SFAs. Specifically, MCFAs can reduce body fat accumulation [10–12] and improve cholesterol metabolism [13–15]. We found that, in contrast to the LCFAs, MCTs (50% C8:0 and 50% C10:0) have a potential for reducing serum LDL-C and TC levels and improving HDL-C levels in hypertriglyceridemic subjects [12, 16]. We also observed that MCTs could ameliorate atherosclerosis in apoE-deficient (apoE^−/−^) mice [17]. Nevertheless, the impact of MCFAs on inflammation and atherosclerosis awaits further investigation.

The Toll-like receptor 4 (TLR4) /nuclear factor kappa B (NF-κB) pathway has significant functions in the stress response and inflammation, and recently it has been suggested to be closely related to human atherosclerosis [18, 19]. TLR4 expression has been observed in smooth muscle cells, vascular endothelial cells, and macrophages [20]. Triggered by TLR4 ligands, it activates the subsequent NF-κB signaling pathway, which increases gene transcription of many pro-inflammatory factors. Moreover, Michelsen et al. have proven that knock out of TLR4 can reduce atherosclerotic lesions in the aorta to a great extent and reduce the levels of circulating pro-inflammatory cytokines in apoE^−/−^ mice [21].

Based on former studies, C8:0 herein is hypothesized to suppress the inflammatory reaction via TLR4/NF-κB signaling and to alleviate the atherosclerotic state in apoE^−/−^ mice. To test it, we carried out a further investigation into the effects of C8:0 on inflammation, mRNA and protein expression of TLR4/NF-κB pathway components, and the atherosclerotic condition of apoE^−/−^ mice. These effects were also investigated in RAW246.7 cells with or without TLR4 knock-down (TLR4-KD).

## Materials & methods

### Materials

MCFAs samples of C8:0, C10:0, as well as LCFAs samples of palmitic acid (C16:0), stearic acid (C18:0), and alpha linolenic acid (C18:3) were obtained from Sigma-Aldrich (St. Louis, MO, USA). Lipopolysaccharide (LPS), DHA, bovine serum albumin (BSA), oil red O, fetal bovine serum (FBS) and DMEM culture medium were provided by Gibco (Grand Island, Nebraska, USA). OCT compound was from Tissue Tek (Sakura, Torrance, CA). Other reagents were available at Sigma-Aldrich.

### Animal experiments

Fifty 4-week-old male apoE^−/−^ mice were obtained from Shanghai Model Organism (License SYXK 2018–0002) and bred in polycarbonate cages (temperature 21–23 °C, humidity 40–60%, 5 animals per cage) on a 12-h light-dark cycle. A basal diet was applied for animal adaptation for more than a week. Then, all the 50 mice (6 weeks old) were randomly divided into 5 groups (*n* = 10): high-fat diet (HFD) with 2% C8:0, HFD with 2% C10:0, HFD with 2% C18:0, HFD with 2% C18:3, and HFD alone. The ingredients list, amount of nutrients, and specific fatty acid compositions of all diets in this work are provided in additional files [see Additional file 1, 2]. Beijing Institute of Nutrition examined the dietary nutrients, and analyzed the dietary lipids with gas-liquid chromatography, as described in detail in our previous report [17].

The body weight and food intake of mice was recorded weekly. Feeding was maintained continuously for 16 weeks, followed by a fasting for more than 8 h (except for water). Subsequently, mice were euthanized via intramuscularly injection with xylazine hydrochloride (10 mg/kg) for blood sampling from the abdominal and collection of tissues for detailed assays.

### Measurement of serum lipid profiles

Commercial kits were employed to test serum TC and triglycerides (TG) (Wako, Osaka, Japan), to detect the level of HDL-C and LDL-C by means of sediment approach (Abcam, Cambridge, UK), and to evaluate the serum level of total bile acid (TBA) (Blue Gene, Shanghai, China). Ratio of HDL-C to LDL-C was subsequently calculated. All the measurements were performed strictly following the instructions from the manufacturer.

### Measurement of inflammatory cytokines in plasma

ELISA kits (R&D Systems, Minneapolis, MN, USA) were utilized to determine the plasma interleukin-1β (IL-1β), interleukin-2 (IL-2), interleukin-6 (IL-6), interleukin-10 (IL-10), monocyte chemoattractant protein-1 (MCP-1) and TNF-α levels following the instructions from the manufacturer.

### Assessment of atherosclerosis in the aorta and aortic sinus

At the end of the study, five mice were randomly chosen from each group to estimate the atherosclerotic plaque areas using a previously reported method [17]. The dissected aorta from the root to the abdominal region was fixed in formalin after a careful removal of all connective tissues. A longitudinal incision was made over the rest of the entire aorta, followed by pinning in a posture with the lumen side up. After staining the aorta with oil red O, digital photographs were taken. Image-Pro Plus 6.0 was used to measure the total surface area and the total oil red O-positive lesion area. The percentage of the lesion areas to the total areas was used to assess the extent of atherosclerotic lesion.

The aortic root tissue frozen in O.C.T. was cut into 10 μm serial sections, and the total oil red O-positive lesion area was measured by NanoZoomer Digital Pathology 2.0 (Hamamatsu, Japan).

### Real-time PCR analysis

To analyze the RNA expression levels, cells were harvested while aorta samples (approximately 50 mg) were rinsed with PBS at low temperature (on ice), followed by homogenization. Total RNA was isolated using TRIzol reagent from Omega Bio-Tek (Norcross, GA, USA), and qRT-PCR analysis was conducted subsequently. The Moloney murine leukemia virus reverse transcriptase (Invitrogen, CA, USA) was employed for cDNA preparation with RNA (1 μg) as the raw material. The obtained cDNA was subjected to qRT-PCR analysis in a fluorometric thermal cycler using the Line-Gene fluorometric PCR detection system (BoRi Technology, China) and the BioEasy Master mix (SYBR Green). The initial denaturation was performed for the reactant mixtures via a 2-min incubation at 95 °C, followed by 45 cycles comprising 95 °C/20 s, 59 °C/25 s, and 72 °C/30 s. The Primer Express 3.0 software was utilized for primer design (Table 1).

**Table 1:** Primer sequences in qRT-PCR

Gene	Sense primer (5′-3′)	Antisense primer (5′-3′)	Accession No.
TLR4	TCAGTTCTCACCTTCCTCCTG	GTTCATTCCTCACCCAGTCTTC	GQ_503242.1
MyD88	GATGGTAGCGGTTGTCTCTGAT	GATGCTGGGGAACTTTCTTC	AB292176.1
NF-κB	AGTACCCTGAGGCTATAACTCGC	TCCGCAATGGAGGAGAAGTC	EU399817.1
TNF-α	TCCAATGGCAGAGTGGGTATG	AGCTGGTTGTCTTTCAGCTTCAC	NM_214022.1
TAK1	GAAGGTGGATCCCTGCACAA	CATGCAAATATGCCAGGCCC	XM_002101880.2
MAPK	GCTGAAGCGCCATTCAAGTT	CCTCTGAGCCCTTGTCCAAT	NM_001357115.1
IKKα	GGCTGGACAGCGTCTCTTTA	GGTGGAAGATGGAGCCAGAC	NM_001044308.2
IKKβ	GTGCCTGTGACAGCTTACCT	ACTGCGTTTGCACTTTTGCT	NM_010546.2
β-Actin	TGAGCTGCGTTTTACACCCT	GCCTTCACCGTTCCAGTTTT	NM_007393.5

*TLR4* toll-like receptor 4, *MyD88* myeloid differentiation primary response 88, *NF-κB* nuclear factor kappa B, *TNF-α* tumor necrosis factor alpha, *TAK1* TGF-β-activated kinase 1, *MAPK* mitogen-activated protein kinase, *IKKα* inhibitor kappa B kinase α, *IKKβ* inhibitor kappa B kinase β

Relative quantification was performed through the ΔCt method, with the difference in Ct between the reference gene (β-actin) and the target gene equaling the ΔCt values for the tested samples. For each sample, target gene expression was normalized according to the eq. 2^−ΔΔCt(2ΔCt(actin) − ΔCt(target gene))^.

### Fatty acid preparation

Fatty acids were prepared referring to the operational details in our previous report [17]. Briefly, stock solutions (20 mmol/L) of C8:0, C16:0, and DHA were procured by dissolving a preset amount of solute in ethanol. The samples were diluted to 2 mmol/L, 1 mmol/L, and 0.5 mmol/L for practical use with cell culture medium containing 20 mg/L endotoxin-free BSA. Before cell addition, the obtained solutions were incubated for 1 h at 37 °C.

### RAW 264.7 cell experiments

The RAW 264.7 cell line was provided by Peking Union Medical College. Cell cultivation was performed in a humidified incubator (95% air, 5% CO_2_, 37 °C) in DMEM supplemented with heat-inactivated FBS (10%), L-glutamine (2 mmol/L), vitamins (1×), and antibiotics (streptomycin, 100 g/L and penicillin, 100 U/mL).

The cells were cultivated for 24 h in 24-well plates at a density of 1.5 × 10^5^ cells/well. The cultivation medium was refreshed with new medium containing LPS (100 ng/mL final concentration) supplemented with C8:0, C16:0, or DHA. Another round of cell incubation was carried out for 12, 24, or 48 h, respectively. The following RAW264.7 treatment groups were included: (1) control; (2) LPS; (3) LPS + C8:0 (50 μmol/L); (4) LPS + C8:0 (100 μmol/L); (5) LPS + C8:0 (200 μmol/L); (6) LPS + C16:0 (100 μmol/L); and (7) LPS + DHA (100 μmol/L). Afterwards, the cells were washed with PBS at low temperature (on ice) three times for media removal, and ELISA kits were employed for measurement of TNF-α, MCP-1, IL-1β and IL-6 in the cell lysates, following the manufacturer’s instructions. These experiments were repeated, and the cells were harvested. Finally, proteins and RNA were isolated to analyse the expression of TLR4, MyD88, NF-κB, and TNF-α.

### TLR4-KD in RAW 264.7 cells

The RAW264.7 cells (1.5 × 10^5^ cells/well) were transfected with a plasmid encoding a TLR4 siRNA (Invivogen, USA) utilizing the Lipofectamine 2000 reagent (Life Technologies, USA) following the manufacturer’s instructions. Then, G418 (500 μg/mL) was employed for isolating stable transfectants after a 3-week selection. A limited dilution method was applied to isolate single clones of stably transfected cells. One stable clone resistant to G418 was kept in G418 (500 μg/mL) -containing medium. DMEM containing 10% FBS was utilized throughout the experimental operation, followed by refreshment with medium containing LPS (100 ng/mL final concentration). The cells were incubated for another 24 h after the addition of C8:0, C16:0, or DHA into the culture medium. The following RAW264.7 cell with TLR4-KD treatment groups were included: (1) control; (2) TLR4-KD; (3) TLR4-KD + LPS; (4) TLR4-KD + LPS + C8:0 (100 μmol/L); (5) TLR4-KD + LPS + C16:0 (100 μmol/L); and (6) TLR4-KD + LPS + DHA (100 μmol/L). Then, ELISA kits were employed to assess the TNF-α, MCP-1, IL-1β and IL-6 levels in the cell lysates, following the instructions from the manufacturer. These experiments were repeated before further analyzing the expression of TLR4, MyD88, NF-κB, and TNF-α by centrifugation and collection of protein and total RNA.

### Western blotting analysis

The cells were cultured for 24 h in 24-well plates at a density of 1.5 × 10^5^ cells/well. The cultivation medium was refreshed with new media containing LPS (100 ng/mL final concentration). Another round of cell incubation was performed for 24 h after the addition of C8:0, C16:0, or DHA. Western blotting analysis of the cultured cells was performed as previously described [22]. Briefly, 30-min harvest and cell lysis were conducted on ice with NP40 cell lysis buffer containing a protease inhibitor cocktail (1×) and phenylmethylsulfonyl fluoride (1 mM). Then, a BCA Protein Assay Kit (Pierce Chemical Company, IL, USA; no. 23225) was employed for evaluation of the protein content in the supernatant. SDS-PAGE was applied to separate 25 μg of protein extract, which was transferred subsequently to PVDF membranes. After an overnight block at 4 °C in TBS-Tween solution containing 5% milk, the membranes were incubated with the primary antibodies for 2 h. The primary antibodies against TLR4 (ab183459, 1:1000), MyD88 (ab2064, 1:1000), NF-κB (ab32360, 1:1000), TNF-α (ab6671, 1:1000), and β-actin (ab6276, 1:5000) were provided by Abcam (Cambridge, MA, USA). Afterwards, the membranes were thoroughly rinsed and subjected to another 2 h of incubation (room temperature) with a HRP-conjugated secondary antibody diluted 1:2000 in blocking buffer. A chemiluminescence detection system (GE Healthcare, Bucks, UK) was employed for observation of protein bands.

### Statistical analysis

Based on a preliminary experiment, the G*Power 3.1.9.2 software (Heinrich-Heine University, Germany) was applied to calculate the minimum sample size required for the detection of a significant difference (*P* < 0.05). A minimum of 10 mice per group was needed for animal experiments. The numbers of samples required for the analysis of the blood lipid profiles, inflammatory cytokines, degree of atherosclerosis in the aorta and aortic sinus and PCR were 10, 10, 5, 5 and 5, respectively. The power calculation indicated a minimum of 6 for the cell experiments. All data in this report are presented as mean ± standard derivation. One-way analysis of variance was performed for data analysis. The independent t-test that decided the statistical significance of differences among various groups as indicated by *P* < 0.05 (two-tailed) was performed with SPSS 19.0 (SPSS, Inc., Chicago, IL, USA).

## Results

### Body weight and food intake of apoE^−/−^ mice

**Fig. 1:**
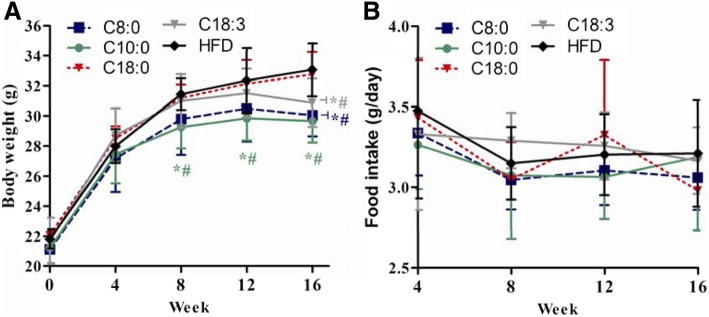
Body weight and food intake of the apoE^−/−^ mice. (**a**) Fasting body weight was determined once per month; (**b**) Food intake during the experiment. Data are mean ± S. D of the mean (*n* = 10). ^*^p < 0.05 versus HFD group; ^#^*p* < 0.05 versus C18:0 group

For both humans and animals, MCTs or MCFAs have been found to suppress the increment of the body fat mass as well as the overall body weight [11, 23, 24]. Our previous study showed similar results in ApoE^−/−^ mice [17]. In this work, the mice in the C10:0 group had much lower body weights than those in the C18:0 and HFD groups after 8 weeks; additionally, the body weight of the mice in the C8:0 and C18:3 groups were significantly lower than those of the C18:0 and HFD groups mice at 16 weeks (Fig. 1a). No significant differences were observed in the average daily food intake (Fig. 1b).

### The serum lipid profiles in the apoE^−/−^ mice

**Fig. 2:**
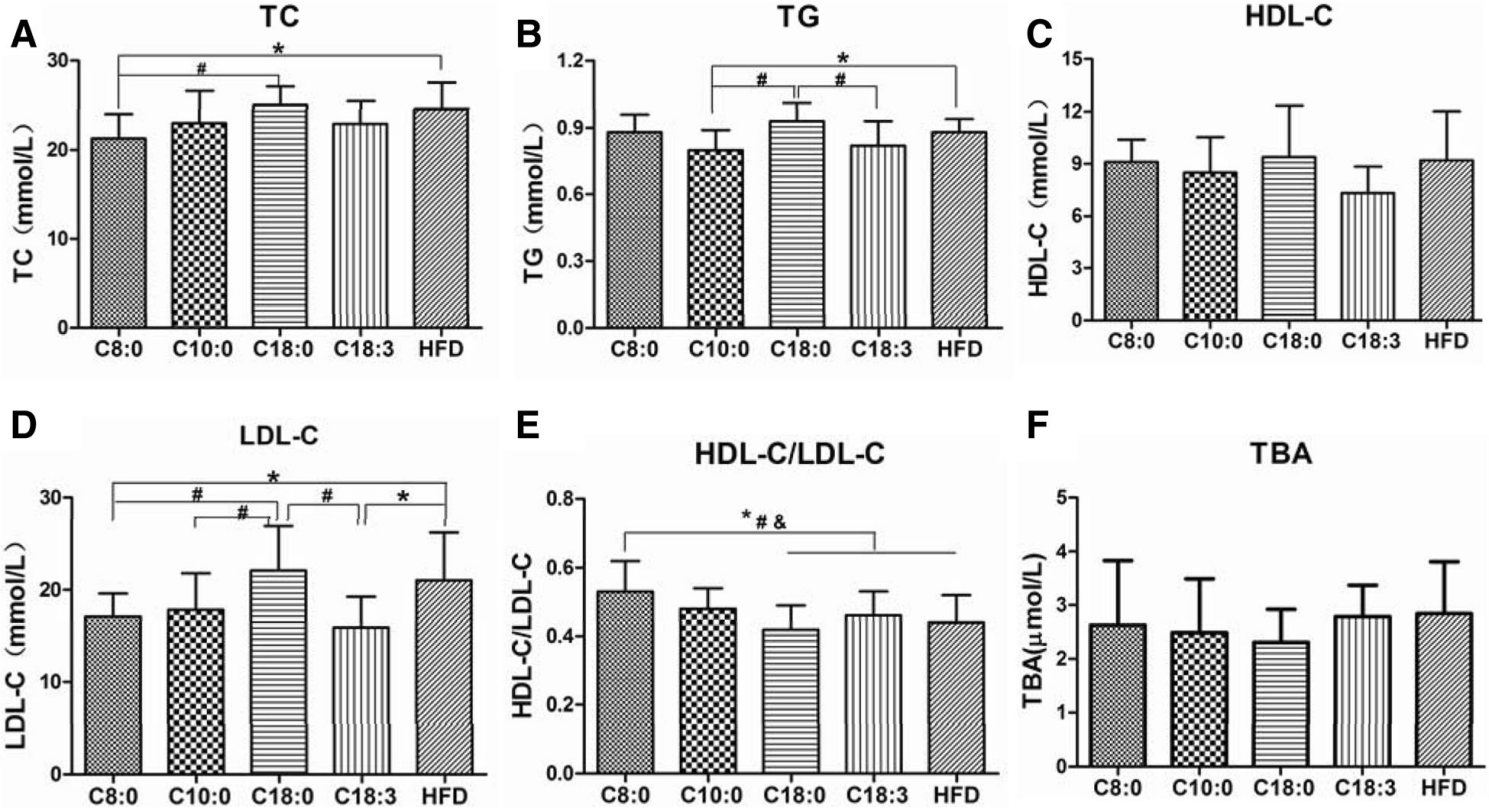
Serum lipid profiles in the apoE^*−/−*^ mice. (**a**) TC, (**b**) TG, (**c**) HDL-C, (**d**) LDL-C, (**e**) HDL-C/LDL-C, (**f**) TBA. Data are mean ± S. D of the mean (*n* = 10). ^*^p < 0.05 versus HFD group; ^#^*p* < 0.05 versus C18:0 group; ^$^*p* < 0.05 versus C18:3 group

Next, we determined the effects of a HFD containing different fatty acids (at 2%) on the serum lipid profiles of the apoE^−/−^ mice after 16 weeks. The C8:0 group mice had a significantly higher HDL-C to LDL-C ratio than the mice in the HFD (1.20-fold), C18:0 (1.26-fold) and C18:3 (1.15-fold) groups (Fig. 2e). The TC (Fig. 2a) and LDL-C (Fig. 2d) levels in the C8:0 group were significantly lower than those in the HFD and C18:0 groups. C8:0 could significantly improve blood lipids, especially the TC and LDL-C levels, which were similar to the findings in the C18:3 group. However, no significant differences were found in the TG, HDL-C, and TBA levels (Fig. 2b, c, and f).

### Serum inflammatory cytokine levels in the apoE^−/−^ mice

**Fig. 3:**
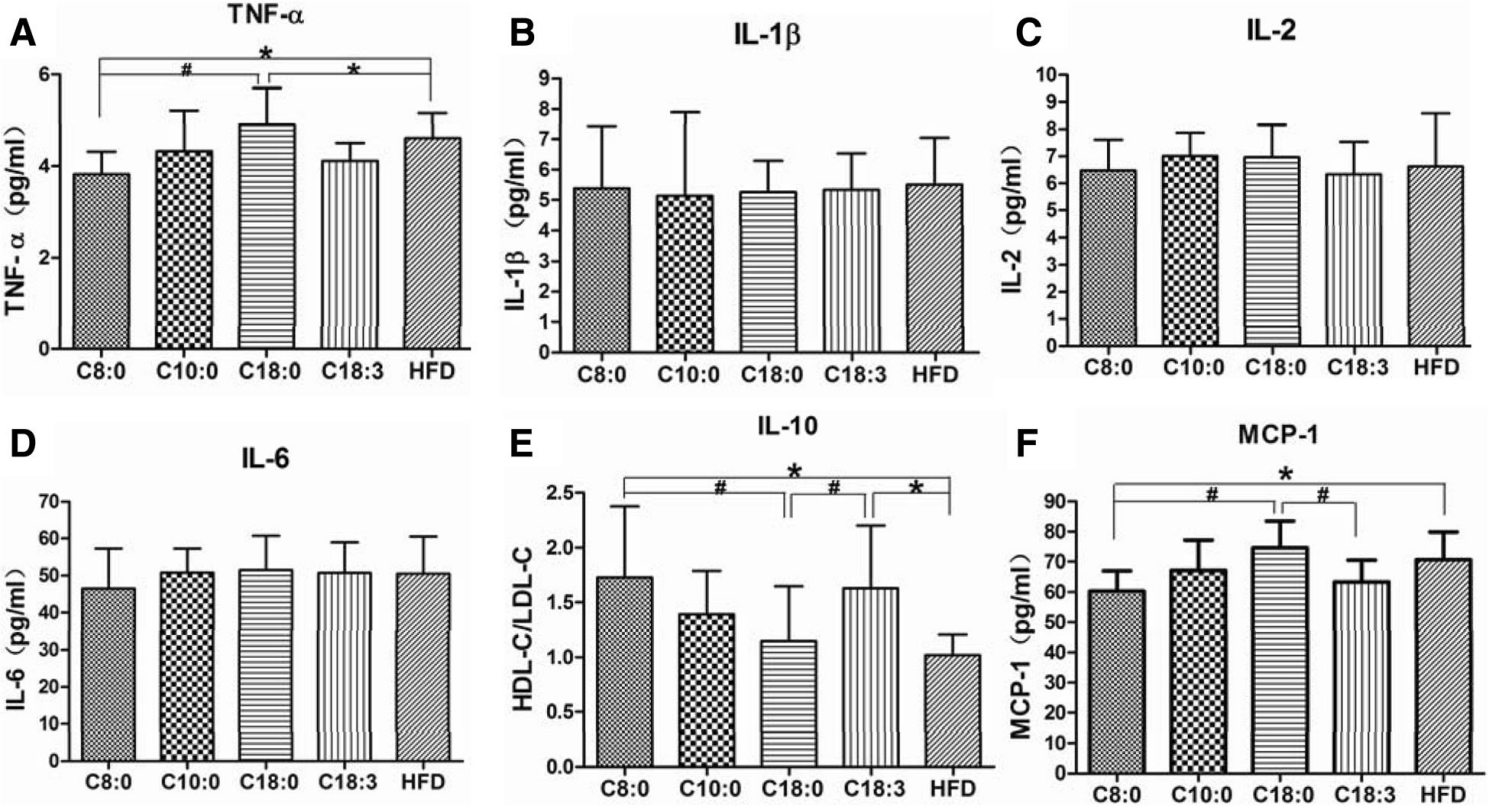
The serum inflammatory cytokine levels in the apoE^−/−^ mice. (**a**) TNF-α, (**b**) IL-1β, (**c**) IL-2, (**d**) IL-6, (**e**) IL-10, (**f**) MCP-1. Data are mean ± S. D of the mean (*n* = 10). ^*^p < 0.05 versus HFD group; ^#^*p* < 0.05 versus C18:0 group

Promoted release of different pro-inflammatory cytokines can be achieved by TLR4/NF-κB signaling activation. According to Fig. 3, the TNF-α (Fig. 3a) and MCP-1 (Fig. 3f) expression levels were lower and IL-10 (Fig. 3e) expression was enhanced in the plasma of the C8:0 group, compared with those of the C18:0 and HFD groups. C8:0 significantly ameliorated inflammation in the apoE^−/−^ mice, which were similar to observations in the C18:3 group.

### The mRNA expression levels of TLR4/NF-κB signaling components in the apoE^−/−^ mouse aortas

**Fig. 4:**
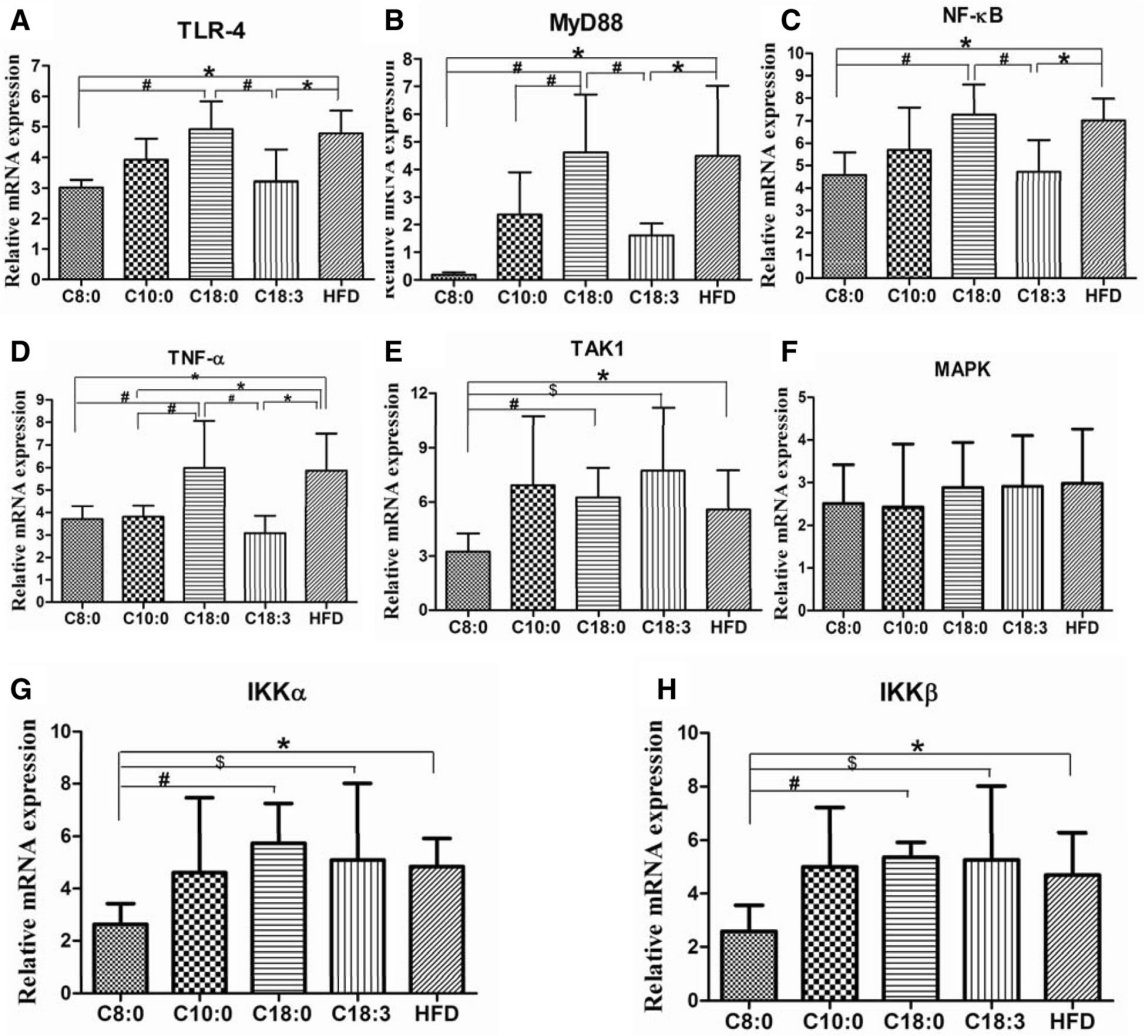
The mRNA expression levels of TLR4/NF-κB signaling components in the apoE^−/−^ mouse aorta. (**a**) TLR4, (**b**) MyD88, (**c**) NF-κB, (**d**) TNF-α, (**e**) TAK1, (**f**) MAPK, (**g**) IKKα, (**h**) IKKβ. Data are mean ± S. D of the mean (*n* = 5). ^*^p < 0.05 versus HFD group; ^#^*p* < 0.05 versus C18:0 group; ^$^*p* < 0.05 versus C18:3 group

Compared with those of the HFD and C18:0 groups, mRNA expression of TLR4, MyD88, NF-κB, TNF-α, TAK1, IKKα, and IKKβ was significantly downregulated in the C8:0 group (Fig. 4). A similar trend was observed for the mRNA expression levels of TLR4, MyD88, NF-κB, and TNF-α in the C18:3 group (Fig. 4). These findings suggested that C8:0 can inhibit the activation of the TLR4/NF-κB signaling pathway in the aorta of apoE^−/−^ mice.

### Atherosclerosis in the aorta and aortic sinus of the apoE^−/−^ mice

**Fig. 5:**
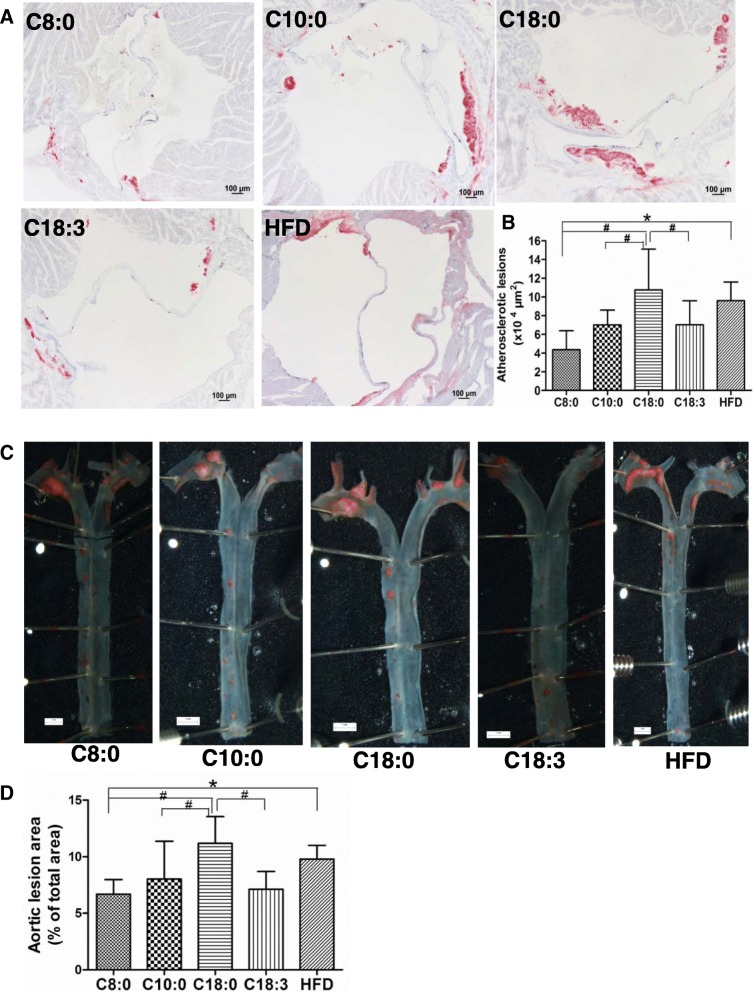
The degree of atherosclerosis in the aorta and aortic sinus in the apoE^−/−^ mice. (**a**) Cross-sections of aortic sinus which were stained by Oil Red O (× 80) (scale bars equal to 100 μm) and (**b**) corresponding quantitative analysis on dye-positive areas. (**c**) Representative photographs of aortic lumen stained by Oil Red O (× 5) (scale bars equal to 1 mm) and (**d**) Quantitative analysis of oil red O-positive areas of the aortic lumen relative to total aortic areas. Data are mean ± S. D of the mean (*n* = 5). ^*^p < 0.05 versus HFD group; ^#^*p* < 0.05 versus C18:0 group

As presented in Fig. 5, a decrease was found in both the cross-sectional aortic sinus and whole aorta atherosclerotic lesions in the C8:0 group, compared with those of the C18:0 group (59 and 40% decreases, respectively) and HFD group (55 and 32% decreases, respectively). In addition, the aortic sinus plaque and whole aorta areas were significantly less in the C10:0 and C18:3 groups than in the C18:0 group (Fig. 5b, d).

### Inflammatory cytokines and TLR4/NF-κB-related gene and protein expression levels in RAW246.7 cells

**Fig. 6:**
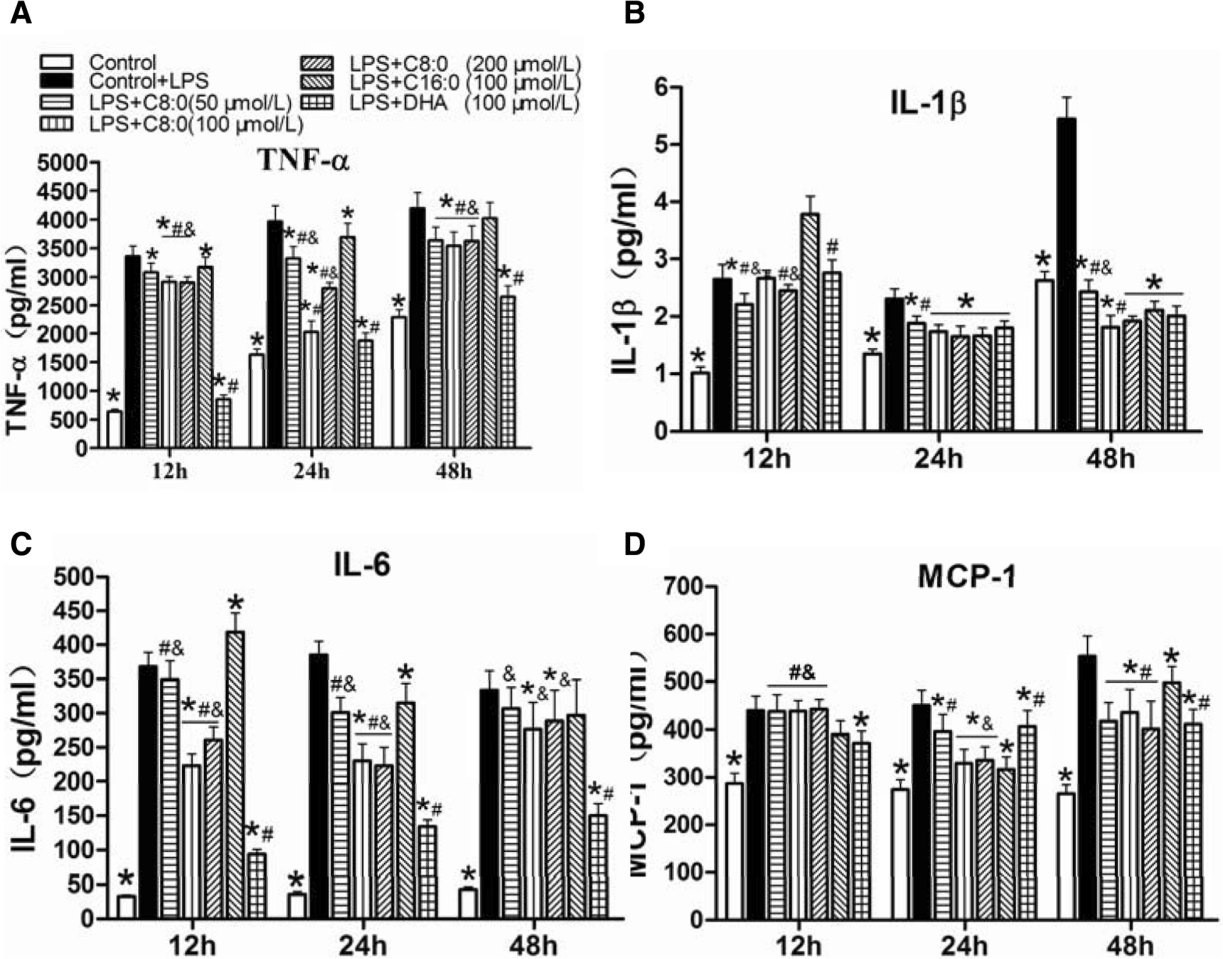
The inflammatory cytokine levels in RAW246.7 cells. (**a**) TNF-α, (**b**) IL-1β, (**c**) IL-6, (D) MCP-1. Data are mean ± S. **d** of the mean (*n* = 6). ^*^p < 0.05 versus LPS group; ^#^*p* < 0.05 versus C16:0 group; ^&^*p* < 0.05 versus DHA

**Fig. 7:**
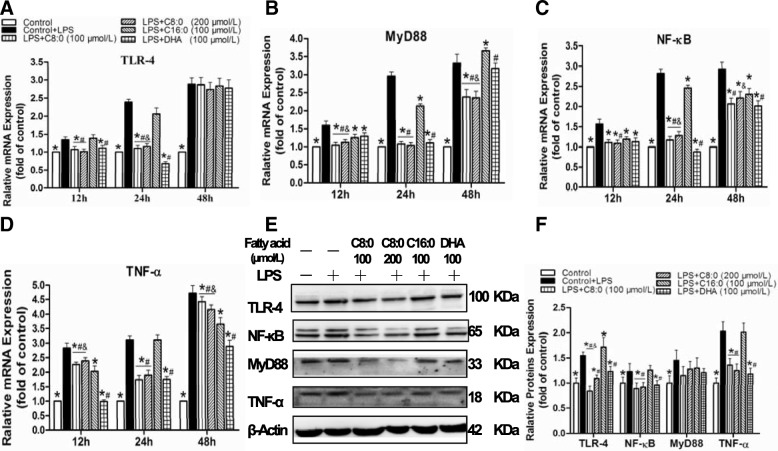
The TLR4/NF-κB-related gene and protein levels in RAW246.7 cells. The mRNA expression levels included (**a**) TLR4; (**b**) MyD88; (**c**) NF-κB; (**d**) TNF-α. Relative protein expression in RAW246.7 cells was obtained from Western blot analysis and β-actin functioned as a control for loading: (**e**) bolt sections and (**f**) gray-scale analysis. Data are mean ± S. D of the mean (*n* = 6). ^*^p < 0.05 versus LPS group; ^#^*p* < 0.05 versus C16:0 group; ^&^*p* < 0.05 versus DHA

The TNF-α (2.4-fold), IL-1β (1.3-fold), IL-6 (10.7-fold), and MCP-1 (1.6-fold) levels (Fig. 6), the TLR4 (2.4-fold), MyD88 (3.0-fold), NF-κB (2.8-fold) and TNF-α(3.1-fold) mRNA expression levels (Fig. 7a-d) and the TLR4 (1.6-fold), MyD88 (1.2-fold), NF-κB (1.5-fold) and TNF-α (2.0-fold) protein expression levels (Fig. 7f) were significantly increased in the LPS-induced RAW246.7 cells than in the control group after 24 h of intervention. Therefore, the influences of various fatty acids on RAW246.7 cells stimulated with LPS were further elucidated. The inhibitory effect of C8:0 on inflammation was most obvious at the 100 μmol/L concentration for a duration of 24 h, The comparison with the LPS groups indicated that the C8:0 group exhibited inhibited production of TNF-α (decreased by 49%) (Fig. 6a), IL-1β (decreased by 25%) (Fig. 6b), IL-6 (decreased by 40%) (Fig. 6c), and MCP-1 (decreased by 26%) (Fig. 6d) and downregulated TLR4 (decreased by 54%) (Fig. 7a), MyD88 (decreased by 64%) (Fig. 7b), NF-κB (decreased by 59%) (Fig. 7c), and TNF-α (decreased by 44%) (Fig. 7d) mRNA expression and TLR4 (decreased by 46%), NF-κB (decreased by 21%), and TNF-α (decreased by 33%) (Fig. 7e, f) protein expression compared with the levels of the C16:0 group (*p* < 0.05). These findings indicated that C8:0 directly affected TLR4-mediated inflammatory responses, which were similar to observations for the DHA group. However, compared with those of DHA, C8:0 significantly increased the TLR4 (Fig. 7a) and NF-κB (Fig. 7c) mRNA expression levels when supplemented at doses of 100 μmol/L and 200 μmol/L but reduced the TLR4 protein expression levels (Fig. 7f) at the 100 μmol/L dose.

### Inflammatory cytokines and TLR4/NF-κB-related gene and protein expression levels in TLR4-KD RAW246.7 cells

**Fig. 8:**
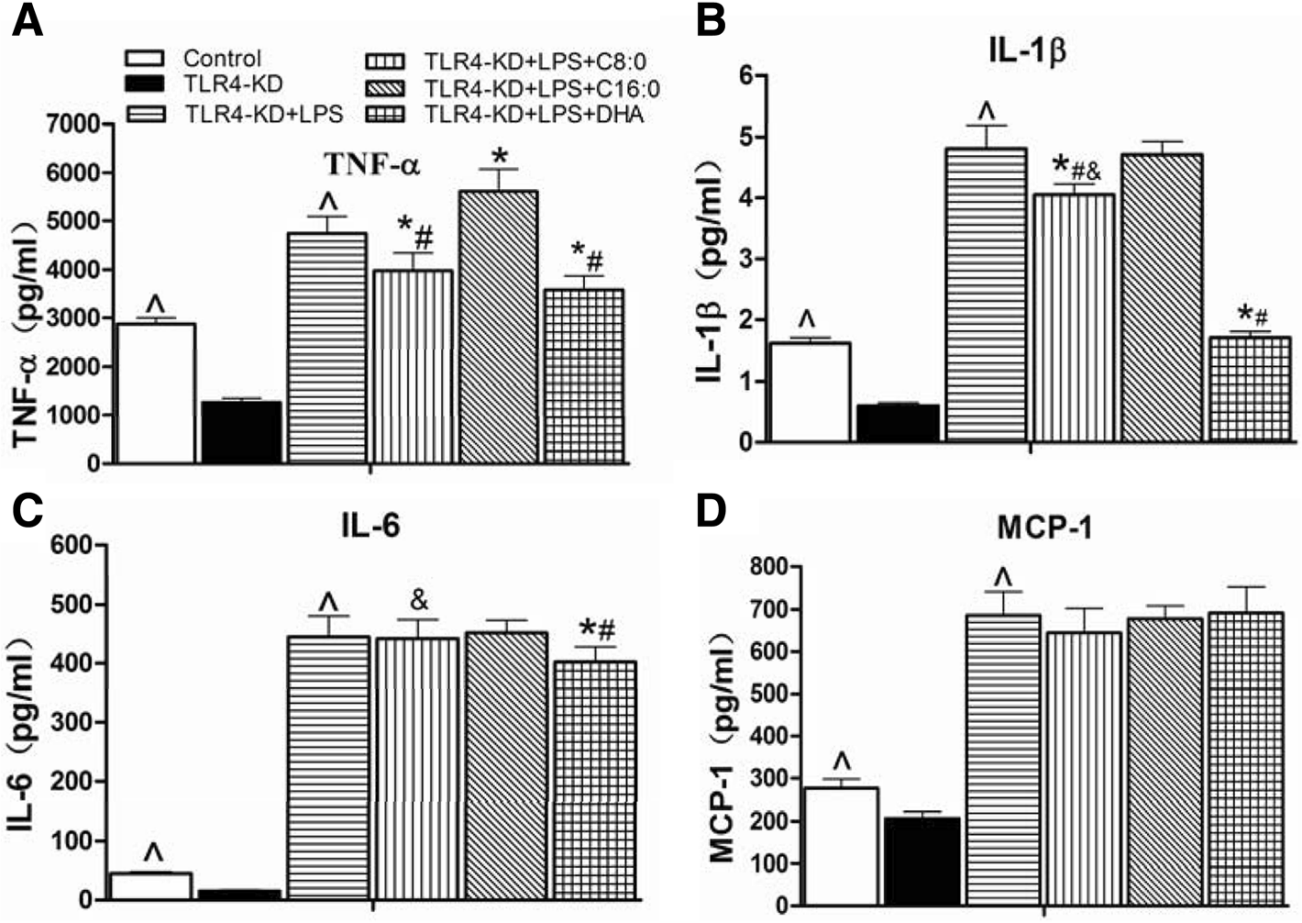
The inflammatory cytokine levels in TLR4-KD RAW246.7 cells. The inflammatory cytokines included (**a**) TNF-α, (**b**) IL-1β, (**c**) IL-6, (**d**) MCP-1. Data are mean ± S. D of the mean (*n* = 6). ^^^*p* < 0.05 versus TLR4-KD group; ^*^p < 0.05 versus TLR4-KD + LPS group; ^#^*p* < 0.05 versus TLR4-KD + LPS + C16:0 group; ^&^*p* < 0.05 versus TLR4-KD + LPS + DHA group

**Fig. 9:**
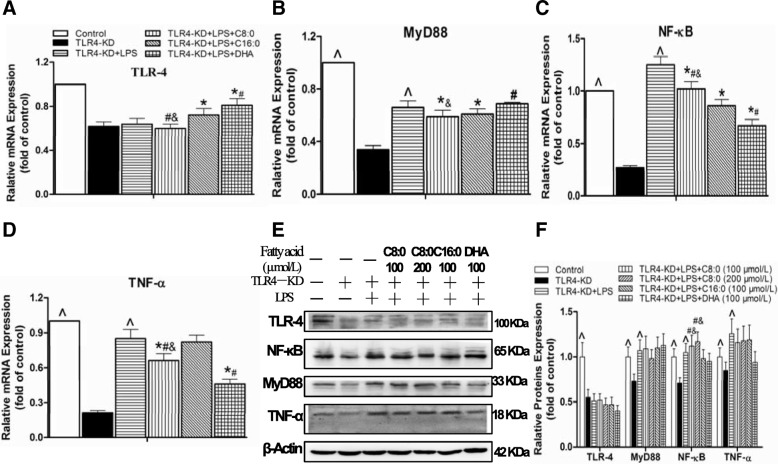
The TLR4/NF-κB-related gene and protein expression levels in TLR4-KD RAW246.7 cells. The mRNA expression levels included (**a**) TLR-4, (**b**) MyD88, (**c**) NF-κB, (**d**) TNF-α. Relative protein expression was obtained from Western blot analysis and β-actin functioned as a control for loading: (**e**) bolt sections and (**f**) gray-scale analysis. Data are mean ± S. D of the mean (*n* = 6). ^^^*p* < 0.05 versus TLR4-KD group; ^*^p < 0.05 versus TLR4-KD + LPS group; ^#^*p* < 0.05 versus TLR4-KD + LPS + C16:0 group; ^&^*p* < 0.05 versus TLR4-KD + LPS + DHA group

The comparison with the control group shown in Figs. 8, 9 suggested that significant reductions occurred in the TLR4-KD group in the inflammatory cytokine levels of TNF-α (decreased by 56%), IL-1β (decreased by 63%), IL-6 (decreased by 67%) and MCP-1 (decreased by 25%), mRNA expression levels of the TLR4 (decreased by 38%), MyD88 (decreased by 66%), NF-κB (decreased by 73%), and TNF-α (decreased by 79%), and protein expression levels of the TLR4 (decreased by 45%), MyD88 (decreased by 27%), NF-κB (decreased by 29%), and TNF-α (decreased by 10%). However, inflammatory cytokine production of TNF-α (3.7-fold), IL-1β (8.0-fold), IL-6 (29.0-fold) and MCP-1 (3.3-fold) (Fig. 8a-d), mRNA expression levels of the MyD88 (1.9-fold), NF-κB (4.6-fold), and TNF-α (4.0-fold) (Fig. 9a-d), and protein expression levels of the MyD88 (1.5-fold), NF-κB (1.5-fold) and TNF-α (1.4-fold) (Fig. 9f) were higher in the TLR4-KD + LPS group than in the TLR4-KD group. We further investigated the effects of different fatty acids on the TLR4-KD + LPS RAW246.7 cells and found that C8:0 at 100 μmol/L had inhibitory effects on TNF-α (decreased by 16%) (Fig. 8a) and IL-1β (decreased by 16%) (Fig. 8b), and downregulated mRNA expression of MyD88 (decreased by 11%) (Fig. 9b), NF-κB (decreased by 18%) (Fig. 9c), and TNF-α (decreased by 22%) (Fig. 9d). Compared to those of the C16:0 group, C8:0 reduced the TNF-α (Fig. 8a) and IL-1β (Fig. 8b) levels and downregulated mRNA expression of TLR4 (Fig. 9a) and TNF-α (Fig. 9d), but up-regulated mRNA and protein expression of NF-κB (Fig. 9e, f). Compared to that of DHA, C8:0 significantly increased the IL-1β (Fig. 8b) and IL-6 (Fig. 8c) levels and downregulated mRNA expression levels of TLR4 (Fig. 9a) and MyD88 (Fig. 9b), but upregulated mRNA expression levels of NF-κB (Fig. 9c) and TNF-α (Fig. 9d). Moreover, the NF-κB (Fig. 9e, f) protein expression level was obviously increased.

## Discussion

Herein, we explored the influences of C8:0 as a dietary supplement on the atherosclerotic lesion areas, serum cholesterol levels, and inflammation in apoE^−/−^ mice compared with mice administered C18:0 and C18:3. The results indicated that the inflammatory cytokine expression was inhibited, plasma lipid profiles were improved, and atherosclerosis was decreased in the presence of C8:0, which was in accordance with our previous observations on these mice [17]. Furthermore, we found that C8:0 suppressed inflammatory reactions through the TLR4/NF-κB signaling pathway.

### C8:0 has positive roles in atherosclerotic alleviation

Many studies have elucidated the intimate relationship between atherosclerosis and fatty acids (either saturated or unsaturated). Omega-3, which is a type of unsaturated fatty acid, may prevent atherosclerotic progression and endothelial dysfunction, and can play a role in the regulation of atherosclerosis when administered as a supplement [25]. However, apart from sporadic reports with conflicting results, the effects of DHA supplementation on atherosclerotic lesion areas have rarely been studied. Dietary DHA can reduce the local expression of the pro-inflammatory cytokine IL-1β in apoE^−/−^ mice [26]. The n-3 polyunsaturated fatty acid can modify the incorporation mode into tissues and inhibit hypoxia-induced atherosclerotic progression in apoE^−/−^ mice [27]. Wang et al. [28] found that 10 weeks of n-3 PUFA fish oil supplementation alleviated atherosclerosis of the aortic root in apoE^−/−^ mice to a significant albeit limited extent. Conversely, some studies showed that supplementing chow with n-3 PUFAs for 14 [29] or 20 weeks [30] had little influence on the development of atherosclerotic lesions in apoE^−/−^ mice. However, a review showed that n-3 PUFAs could prevent atherosclerotic morbidity, and evidence suggested that this effect might be mediated by improving endothelial dysfunction [31]. By surpassing the effects of oxidation and inflammatory stress, fats rich in linoleic acid can prevent atherosclerotic progression in apoE^−/−^mice, compared to those fed a diet rich in saturated fatty acids [32]. Substituting excessive dietary SFAs for other types of macronutrients can contribute to early induction of atherosclerosis in animals [33]. Varying results have been reported in a great number of promising studies on the correlation of SFA intake with CVD [34]. Unlike LCFAs (> 12 carbons), MCFAs can undergo rapid gastrointestinal hydrolysis and absorption, be directly transported via the portal veins, and undergo fast β-oxidation in the liver [35]. This metabolic specificity, which leads to enhanced catabolism but reduced tissue storage [36], was in relation to the physiological effects exerted by dietary MCFAs. It is reported to be beneficial or neutral compared to the metabolism of LCFAs. Some research also demonstrated that MCTs or MCFAs could play a positive role in reducing serum LDL-C and TC not only in mice [37, 38] but also in humans [12, 15, 16, 39]. C8:0 is a type of MCFA, which we found could significantly reduce TC and LDL-C, compared to palmitic acid or stearic acid in apoE^−/−^ mice [38]. C8:0 can also improve the HDL-C to LDL-C ratio and reduce the atherosclerotic extent in the aorta and aortic sinus [17]. These results suggested that C8:0 was similar to omega-3 in terms of its positive roles in atherosclerotic alleviation.

### C8:0 inhibits inflammation

As a chronic inflammatory disease, atherosclerosis progresses under the regulation of a large number of modulators, such as cytokines, eicosanoids [40], and dietary fatty acids, which represent another critical family of regulators [41]. SFAs [3, 4] and PUFAs, particularly n-6 PUFA [5], possess pro-inflammatory properties, as indicated by many studies performed both in vivo and in vitro. In contrast, n-3 PUFA, such as EPA and DHA, can exert anti-inflammatory effects [42, 43]. MCFAs or MCTs can ameliorate inflammation. Bertevello et al. [44] achieved improvement in the colon cytokine response and damage reduction in model rats with colitis by partially replacing the n-6 fatty acids with MCTs. Papada et al. [45] reported the anti-inflammatory performance of a diet rich in MCTs in model rats carrying TNBS-induced colitis, in which the IL-6, IL-8, and intercellular adhesion molecule-1 levels were decreased and glutathione S-transferase activity was reduced. Moreover, for a model rat carrying sepsis, an MCT diet could significantly reduce the expression levels of pro-inflammatory cytokines and chemokines (TNF-α, IL-18, macrophage inflammatory protein-2, and MCP-1) in the ileum and Peyer’s patches [46]. Herein, C8:0 has been proven to be prominently efficient in inhibiting inflammatory cytokine expression (i.e., TNF-α and MCP-1) in the plasma, and increasing the IL-10 level compared with those of the group treated with C18:0. However, a minor discrepancy was noted between the C8:0 and C18:3 groups. One meta-analysis reported that C18:3 administration had great therapeutic potential via decreasing patients’ inflammatory markers (e.g., C-reactive protein, IL-6, and TNF-α) that were associated with metabolic syndrome and related diseases [47]. Samantha et al. [48] also found that C18:3 might function to alleviate the inflammatory states of M1-like macrophages via a special pathway that was different from those associated with EPA and DHA. Martínez-Micaelo et al. [49] confirmed that SFAs activated the nod-like receptor protein 3 inflammasome and stimulated IL-1β secretion, whereas DHA (n-3 PUFA) functioned more positively than arachidonic acid in terms of inhibition of inflammasome activation. In vitro studies showed that levels of the inflammatory cytokines TNF-α and IL-1β increased with prolongation of LPS stimulation, compared with those of control group, possibly due to increased cell stress and apoptosis. However, C8:0 inhibited production of TNF-α, IL-1β, IL-6, and MCP-1 in RAW246.7 cells activated by LPS, and the effects were significantly greater when treated with 100 μmol/L and 24 h. Moreover, these observations were similar to those for the DHA group. The effects of MCFAs have also been discussed recently. Tanaka et al. [50] reported the potentiation of C10:0 on IL-8 production in Caco-2 cells stimulated by IL-1β. Hoshimoto et al. [51] first discovered that IL-8 secretion from Caco-2 cells could be suppressed by C8:0 as well as medium-chain C8 triglycerides. These results suggested that C8:0, but not C10:0, inhibited inflammation. However, the mechanisms through which C8:0 dampens inflammation remain unclear.

### C8:0 can suppress inflammation via TLR4/NF-κB signaling

Several studies have confirmed that inflammatory responses mediated by TLR4 may impose significant effects on the initiation and subsequent progression of atherosclerosis [21, 52]. We noted previously that MCTs could ameliorate atherosclerosis via promotion of reverse cholesterol transport [17], which increased the ease of cholesterol export from peripheral cells and prevented intracellular cholesterol accumulation. In this study, we found that C8:0, which is a member of the MCFAs, could suppress inflammatory signaling via the TLR4/NF-κB pathway and improve atherosclerosis in apoE^−/−^ mice. TLRs can regulate both sterile inflammation and that induced by infection via endogenous molecules. Upon LPS binding, the TLR4/CD14/LBP receptor complex engages MyD88 to initiate a downstream signaling cascade, thereby triggering NF-κB and activating genes that encode pro-inflammatory factors, such as cytokines and COX2 [53]. Numerous reports have investigated the stimulatory effects of SFAs on inflammatory responses via a TLR4-involved pathway. In particular, the stimulatory effects of the SFAs C12:0, C16:0, and C18:0 can enhance expression levels of the IL-6 gene in macrophages through this approach [54]. In accordance, MCP-1 expression can also be enhanced by C18:0 via TLR4 [55]. In this study, we showed that C8:0 significantly downregulated the mRNA expression levels of TLR4, MyD88, NF-κB, TNF-α, TAK1, IKKα, and IKKβ compared to those induced by C18:0 in the aortas of apoE^−/−^ mice. Macrophages are the most abundant immune cell type and primary inflammatory cells in atherosclerotic lesions and have an essential role during all stages of atherosclerosis [56]. Excessive lipid accumulation in macrophages, also known as foam cell formation, is a key process during the development of atherosclerosis, leading to vascular inflammation and plaque growth. The expression of TLR4 has been found in macrophages and endothelial cells within human and mouse atherosclerotic lesions; and the TLR4 deficiency significantly reduces the in vivo rate of macrophage lipid accumulation in vascular lesions [57]. The inflammatory responses mediated by TLR4 play important roles in the initiation and progression of atherosclerosis. Therefore, we further observed the effect of C8:0 on the TLR4/NF-κB pathway of macrophages. The assay results obtained from RAW246.7 cells were consistent. Our data showed an altered effect of C8:0 on TLR4 even as a saturated fatty acid, which was different from that of the LCFAs. Furthermore, two pathways have been proposed to underlie the mechanism of SFA-mediated inflammation (i.e., a TLR4-dependent one and a TLR4-independent pathway) [58, 59]. Whereas SFAs can stimulate TLR4 signaling, EPA and DHA have been suggested to play inhibitory roles [60]. The first possibility is that EPA and DHA bind to G protein-coupled receptor 120, thereby inhibiting TAK1 and preventing the downstream NF-κB and JNK signaling pathways [6]. Other explanations for the anti-inflammatory roles played by EPA and DHA include altered phospholipid fatty acid compositions in cell membranes, lipid rafts damage, downregulation of nicotinamide adenine dinucleotide phosphate-oxidase production, upregulation of PPARγ activation, and inhibition of activation of NF-κB, which is a pro-inflammatory factor for transcription [58]. Although SFAs can activate TLR4, the polyunsaturated fatty acids, especially DHA and EPA, may exert opposite effects [60]. We attempted to clarify the mechanism through which C8:0 suppressed the inflammatory reaction via TLR4/NF-κB signaling. In TLR4-KD RAW264.7 cells, it functioned adversely to the effects of C8:0 on inflammatory cytokines; and the effects were different from those observed without TLR4-KD. We also found a remarkable upregulation effect of C8:0 on the NF-κB mRNA and protein expression levels compared to those detected in the presence of C16:0. Despite the downregulation of TLR4 and MyD88 mRNA expression induced by C8:0, it significantly strengthened TNF-α and IL-1β expression, increased TNF-α and NF-κB mRNA expression, and elevated NF-κB expression compared with those obtained in the presence of DHA. These results indicated that C8:0 might affect TLR4-mediated inflammatory responses. However, the mechanism through which C8:0 inhibits TLR4 is not clearly understood. Therefore, further research is needed to determine this mechanism.

### Implications and limitations

There are some strengths of our study. Firstly, MCTs, mainly containing saturated fatty acids, can inhibit inflammation in ApoE^−/−^ mice. Secondly, that anti-inflammatory effect of medium chain fatty acids is mainly C8:0, but not C10:0 in ApoE^−/−^ mice. Last, this work should be the first case to indicate that C8:0-mediated inhibition of TLR4/NF-κB signaling may improve atherosclerosis. It provides a theoretical basis for the prevention of chronic inflammatory diseases by C8:0.

This study had some limitations. (1) We should have examined whether C8:0 had any effects on inflammation and atherosclerosis in TLR4^−/−^ ApoE-KO mice or in ApoE^−/−^ mice treated with a TLR4 siRNA. Additionally, these experiments do not indicate that the same effects will occur in humans. Therefore, further clinical studies are warranted. (2) Research on the mechanisms through which C8:0 regulates TLR4 is limited. The specific protein-coupled receptor to which C8:0 binds and the mechanism through which C8:0 binds TLR4 are not known. Although C8:0 can inhibit the TLR4/NF-κB signaling pathways to suppress inflammation, this inhibition occurs through MyD88-dependent and/or MyD88-independent pathways is uncertain. (3) TLR4 expression has been found to be broad in many cell types in the vessel wall that are related to atherosclerotic pathogenesis. However, the evaluation of how C8:0 affected TLR4 expression in both endothelial and vascular smooth muscle cells on the vessel wall was insufficient in this work. (4) Other probable mechanisms may exist besides the TLR4/NF-κB inhibition that underlies the C8:0-mediated dampening of the inflammatory response and improvement of atherosclerosis.

## Conclusion

**Fig. 10:**
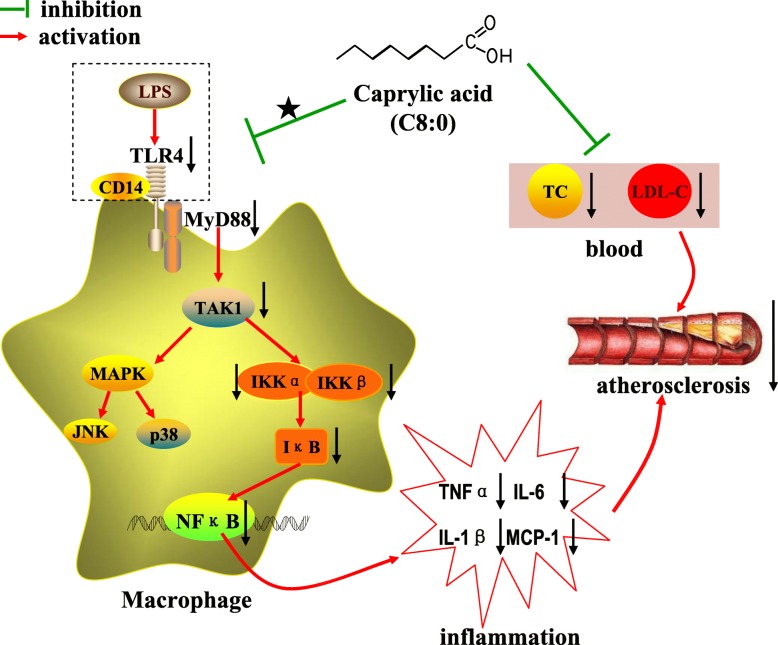
The proposed mechanism underlying the effects of C8:0 on inhibiting inflammation and reducing the atherosclerotic. Arrows (↓) represent a decreasing level and downregulation of activity or protein or mRNA expression. The“★” indicate the manner in which C8:0 acts on TLR4 or macrophage is unclear. LPS, lipopolysaccharide; TLR4, toll-like receptor 4; MyD88, myeloid differentiation primary response 88; NF-κB, nuclear factor kappa B; TNF-α, tumor necrosis factor alpha; TAK1, TGF-β-activated kinase 1; MAPK, mitogen-activated protein kinase; JNK, c-Jun N-terminal kinase; IKKα, inhibitor kappa B kinase α; IKKβ, inhibitor kappa B kinase β; IL-1β, interleukin-1β; IL-6, interleukin-6; MCP-1, monocyte chemoattractant protein-1; TC, total cholesterol; LDL-C, low density lipoprotein cholesterol

Our results suggested that C8:0 supplementation confers protection against atherosclerosis through suppression of inflammation and improvement of blood lipids. The anti-inflammatory efficacy of C8:0 was mediated by downregulation of TLR4 and suppression of the NF-κB signaling pathways (Fig. 10). These findings indicated that C8:0 may be a potential candidate for ameliorating and preventing atherosclerosis and related chronic inflammatory diseases.

## Additional files

Additional file 1: Compositions of applied diets (g/kg). (PDF 24 kb)

Additional file 2: Compositions of fatty acids in the applied diets (g/kg). (PDF 7 kb)

## Abbreviations

ApoE^−/−^: ApoE-deficient
DHA: Docosahexenoic acid
DMEM: Dulbecco’s modified Eagle medium
EPA: Eicosapentaenoic acid
HDL-C: High density lipoprotein cholesterol
HFD: High-fat diet
IKKα: Inhibitor of nuclear factor kappa-B kinase α
IKKβ: Inhibitor of nuclear factor kappa-B kinase β
IL-10: Interleukin-10
IL-1β: Interleukin-1β
IL-2: Interleukin-2
IL-6: Interleukin-6
LCFAs: Long-chain saturated fatty acids
LDL-C: Low density lipoprotein cholesterol
LPS: Lipopolysaccharide
MAPK: Mitogen-activated protein kinase
MCFAs: Medium-chain fatty acids
MCP-1: Monocyte chemoattractant protein-1
MCTs: Medium-chain triglycerides
MyD88: Myeloid differentiation primary response gene 88
NF-κB: Nuclear factor kappa B
PCR: Polymerase chain reaction
PUFA: Polyunsaturated fatty acids
SFAs: Saturated fatty acids
TAK1: Transforming growth factor activated kinase-1
TC: Total cholesterol
TG: Triglyceride
TLR4: Toll-like receptor 4
TLR4-KD: TLR4 knock-down
TNF-α: Tumor necrosis factor α
C8:0: Caprylic acid
C10:0: Capric acid
C16:0: Palmitic acid
C18:0: Stearic acid
C18:1: Oleic acid
C18:3: Linolenic acid
