# Supplementary material for: Caprylic acid suppresses inflammation via TLR4/NF-κB signaling and improves atherosclerosis in ApoE-deficient mice
Source: Nutr Metab (Lond). 2019 Jun 6;16:40. doi: 10.1186/s12986-019-0359-2 (PMC6555760; doi:10.1186/s12986-019-0359-2)
Supplement: Supplementary file 1 — Compositions of applied diets (g/kg). (PDF 24 kb) [file 12986_2019_359_MOESM1_ESM.pdf]

Table 1 Compositions of applied diets (g/kg)

Ingredients	C8:0 diet	C10:0 diet	C18:0 diet	C18:3 diet	HFD diet
Corn starch	308.80	308.80	308.80	308.80	315.10
Casein	155.40	155.40	155.40	155.40	158.60
L-Cystine	2.33	2.33	2.33	2.33	2.38
Fiber(cellulose)	38.85	38.85	38.85	38.85	39.64
Sucrose	77.70	77.70	77.70	77.70	79.29
Maltose dextrin	102.60	102.60	102.60	102.60	104.69
AIN mineral mix 93G	27.20	27.20	27.20	27.20	27.76
AIN vitamin mix 93	7.77	7.77	7.77	7.77	7.93
Choline bitartrate	1.94	1.94	1.94	1.94	1.98
Soybean oil	54.39	54.39	54.39	54.39	55.50
Tert-butylhydroquinone	0.01	0.01	0.01	0.01	0.01
Lard	100.00	100.00	100.00	100.00	102.04
Yolk powder	100.00	100.00	100.00	100.00	102.04
Bile salt	3.00	3.00	3.00	3.00	3.06
C8:0	20.0	0.00	0.00	0.00	0.00
C10:0	0.00	20.0	0.00	0.00	0.00
C18:0	0.00	0.00	20.0	0.00	0.00
C18:3	0.00	0.00	0.00	20.0	0.00
Energy (kJ/g)	18.44	18.44	18.44	18.44	18.05
Percentage of nutrient (%)					
Protein	18.01	18.01	18.01	18.01	18.38
Fat	19.42	19.42	19.42	19.42	17.78
Carbohydrate	48.47	48.47	48.47	48.47	49.46
Cholesterol	0.029	0.029	0.029	0.029	0.030
Mineral mixture	0.078	0.078	0.078	0.078	0.080
Vitamin mixture	0.052	0.052	0.052	0.052	0.053
Fiber	0.150	0.150	0.150	0.150	0.153
Water	0.950	0.950	0.950	0.950	0.969
Others	0.106	0.106	0.106	0.106	0.108

Abbreviations: caprylic acid (C8:0), capric acid (C10:0), stearic acid (C18:0), linolenic acid (C18:3), and high-fat diet (HFD).
