# Supplementary material for: Caprylic acid suppresses inflammation via TLR4/NF-κB signaling and improves atherosclerosis in ApoE-deficient mice
Source: Nutr Metab (Lond). 2019 Jun 6;16:40. doi: 10.1186/s12986-019-0359-2 (PMC6555760; doi:10.1186/s12986-019-0359-2)
Supplement: Supplementary file 2 — Compositions of fatty acids in the applied diets (g/kg). (PDF 7 kb) [file 12986_2019_359_MOESM2_ESM.pdf]

Table 2 Compositions of fatty acids in the applied diets (g/kg)

Ingredients	C8:0 diet	C10:0 diet	C18:0 diet	C18:3 diet	HFD diet
C8:0	20.45	ND[a]	ND[a]	ND[a]	ND[a]
C10:0	ND[a]	19.85	ND[a]	ND[a]	ND[a]
C14:0	1.84	1.84	1.84	1.84	1.88
C16:0	43.36	43.36	43.36	43.36	44.24
C16:1	3.78	3.78	3.78	3.78	3.86
C18:0	21.88	21.88	41.88	21.88	22.33
C18:1	64.02	64.02	64.02	64.02	65.33
C18:2	34.87	34.87	34.87	34.87	35.58
C18:3	1.94	1.94	1.94	21.94	1.98
C20:0	0.41	0.41	0.41	0.41	0.42
C20:4	0.82	0.82	0.82	0.82	0.84
C22:0	0.31	0.31	0.31	0.31	0.32
C22:6	0.51	0.51	0.51	0.51	0.52

[a]ND for not detectable
